# Can Magnetic Resonance Imaging Reveal Lower Motor Neuron Damage after Traumatic Spinal Cord Injury? A Scoping Review

**DOI:** 10.1089/neur.2021.0019

**Published:** 2021-11-29

**Authors:** Jethro Moneo, John L.K. Kramer, Thomas E. Nightingale, Michael J. Berger

**Affiliations:** ^1^ MD Program, Faculty of Medicine, Department of Medicine, University of British Columbia, Vancouver, British Columbia, Canada.; ^2^ International Collaboration on Repair Discoveries (ICORD), Vancouver, British Columbia, Canada.; ^3^ School of Kinesiology, Department of Medicine, University of British Columbia, Vancouver, British Columbia, Canada.; ^4^ Division of Physical Medicine and Rehabilitation, Department of Medicine, University of British Columbia, Vancouver, British Columbia, Canada.; ^5^ School of Sport, Exercise and Rehabilitation Sciences, University of Birmingham, Birmingham, United Kingdom.

**Keywords:** diffusion tensor imaging, lower motor neuron, magnetic resonance imaging, nerve transfer surgery, spinal cord injury

## Abstract

Restoring muscle function to patients with spinal cord injuries (SCIs) will invariably require a functioning lower motor neuron (LMN). As techniques such as nerve transfer surgery emerge, characterizing the extent of LMN damage associated with SCIs becomes clinically important. Current methods of LMN diagnosis have inherent limitations that could potentially be overcome by the development of magnetic resonance imaging (MRI) biomarkers: specific features on MRI that are indicative of LMN integrity. To identify research on MRI biomarkers of LMN damage in the acute phase after SCI, we searched PubMed, EMBASE, MEDLINE, and the Cochrane Central Register of Controlled Trials for articles published from inception to April 27, 2021. Overall, 2 of 58 unique articles screened met our inclusion criteria, both of which were small studies. We therefore identify MRI biomarkers of LMN damage overlying SCI as a notable gap in the literature. Because of the lack of existing literature on this specific problem, we further our discussion by examining concepts explored in research characterizing MRI biomarkers of spinal cord and neuronal damage in different contexts that may provide value in future work to identify a biomarker for LMN damage in SCI. We conclude that MRI biomarkers of LMN damage in SCI is an underexplored, but promising, area of research as emerging, function-restoring therapies requiring this information continue to advance.

## Introduction

**FIG. 1.**
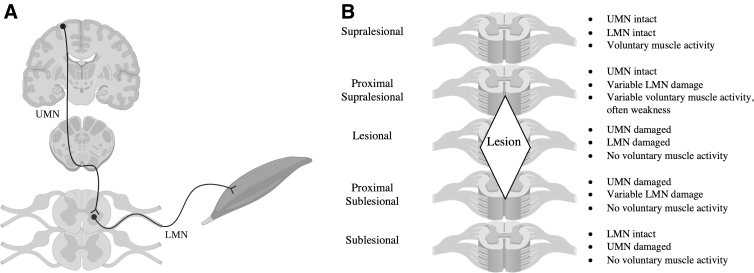
(**A**) The pathway for voluntary muscle control involves signal transmission from the motor cortex by upper motor neurons (UMNs), which decussate at the medulla of the brainstem, before traveling down the spinal cord and synapsing with a lower motor neuron (LMN), that then travels peripherally to innervate a skeletal muscle. (**B**) The viability of UMNs and LMNs is variable at the lesional and perilesional segments in a traumatic spinal cord injury. Above the lesion site (supralesional), all components of the pathway are intact and voluntary muscle activity is unchanged. Below the lesion site (sublesional), the UMN is damaged or has lost its supraspinal input, but the LMN pathways are intact. At the lesion site, both the UMN and LMNs are damaged. In the regions directly adjacent to the lesion site (caudal supralesional and cranial sublesional), there may be variable UMN and LMN integrity. Adapted from: *Archives of Physical Medicine and Rehabilitation*, Vol. 97/6, Anne M. Bryden, Harry A. Hoyen, Michael W. Keith, Melvin Mejia, Kevin L. Kilgore, and Gregory A. Nemunaitis, Upper extremity assessment in tetraplegia: the importance of differentiating between upper and lower motor neuron paralysis, pages S97–S104, copyright (2016), with permission from Elsevier.

Voluntary muscle movement requires an intact neuronal pathway in which cortical inputs travel down the spinal cord as upper motor neurons (UMNs), which synapse onto lower motor neurons (LMNs) in the ventral horn at a specific vertebral level, before exiting the cord to travel peripherally to the target muscle (Fig. 1A). Spinal cord injuries (SCIs) are typically conceptualized as damage to the UMN pathways; however, superimposed LMN damage at or within several segments of the neurological level of injury often occurs.^1–3^ The integrity of LMNs is not routinely assessed, but is critical to the success of emerging treatments with the potential to restore meaningful functions to patients, such as nerve transfer surgery.^4,5^ The timeline for restoring LMN function is limited by irreversible atrophy and fibrosis that occur in muscle after 12–18 months of denervation.^6^ Electrodiagnosis is currently the common method for diagnosis of LMN damage, but is not possible until Wallerian degeneration progresses sufficiently (i.e., 3–4 weeks after injury).^7^

Further, the early period after SCI is one of great uncertainty for clinicians and patients where the focus is on lifesaving measures, mitigating secondary complications of injury, and conveying information about prognosis. To facilitate improved diagnosis of LMN damage after SCI, we conducted a scoping review to identify existing research involving the use of conventional or quantitative magnetic resonance imaging (MRI) features as biomarkers of LMN damage in SCI. To inform this discussion, we provide a a brief overview of the pathophysiology of LMN damage in SCI, clinical relevance of characterizing LMN damage, and rationale for developing MRI biomarkers.

### Pathophysiology of lower motor neuron damage in spinal cord injury

Coulet and colleagues^1^ were the first to incorporate LMN viability into a conceptual framework of muscle paralysis in SCI. They described three levels of SCI; the functional segment above the lesion; the sublesional cord, with paralysis resulting from damaged corticospinal inputs, despite intact LMNs; and the segment of spinal cord and associated nerve roots (termed the “injured metamere”) at the level of the lesion, with combined UMN and LMN damage underlying paralysis. Within the injured metamere, patterns of LMN integrity were variable because of the heterogeneous nature of trauma in SCI, causing differences in the longitudinal and axial extent of damage. Bryden and colleagues^8^ subsequently further stratified this concept by adding proximal supra and sublesional segments that possess variable amounts of LMN viability (Fig. 1B).

SCI can damage LMNs at any point along their course. Direct intraspinal anterior horn damage from the initial contusion, laceration, or compression injury can lead to apoptotic, necrotic, or excitotoxic death of the anterior horn cells.^9^ Endothelial cell death of blood vessels causes hemorrhage, which disrupts the oxygen and nutrient supply to the anterior horn cells, followed by an inflammatory response that leads to edema.^10^ Outside the spinal cord, LMN damage can result from compression, laceration, or avulsion of the ventral root, spinal nerve, or brachial plexus attributable to associated vertebral fracture or ligamental tearing.^11–13^ Finally, damage to peripheral nerves is common in patients with SCI attributable to chronic entrapment from wheelchair use and repetitive transfers (e.g., carpal tunnel syndrome).^14,15^

### Clinical relevance of lower motor neuron integrity

Emergence of nerve transfer surgery highlights a critical role for assessing LMN damage in patients with SCI.^5,16^ Nerve transfers involve coapting a relatively expendable nerve or nerve fascicle from above the spinal cord lesion site to an LMN exiting the spinal cord below the lesion, thereby restoring voluntary control to a functionally important muscle group.^5^ Nerve transfers have several key advantages compared to more commonly used tendon transfer procedures: They do not require long periods of post-operative immobilization, do not alter the biomechanics of the repaired muscle, and may allow for nuanced motor control.^17^ The caveat to this procedure is that the recipient LMN must be intact, or else the procedure must be performed before the denervated muscles undergo irreversible atrophy and fibrosis after 12–18 months.^6^ If the LMN is intact, the time frame of nerve transfer is theoretically unlimited, with a report of successful nerve transfer 13 years after SCI.^18^ Beyond nerve transfer procedures, an intact LMN is essential for any attempts at curative therapy or rehabilitation efforts that aim to restore muscle strength. Determining the extent of LMN injury is therefore crucial for optimal decision making in therapies with the potential to meaningfully improve function and quality of life.

### Current methods of lower motor neuron assessment

Formalized LMN testing in SCI is often overlooked clinically. Paralysis is defined as such without investigation into the cause, be it UMN, LMN, or peripheral nerve, given that there has historically been little that can be done with this information.^19^ Additionally, managing the complex pathophysiological, psychosocial, and medical aspects of such a life-altering event in a timely and patient-centered manner can leave both physician and patient with little desire for complex and specialized LMN-diagnostic procedures. However, as the clinical importance of characterizing the lesion type grows, serial evaluation of the LMN will likely be warranted. Currently, electrodiagnosis is the most common technique to diagnose LMN damage and usually includes semiquantitative needle electromyography (EMG), in which the electrical activity of muscles is observed to detect patterns associated with denervation, and nerve conduction studies, in which the integrity of neurons is tested by stimulating the neurons and recording over nerves/muscles of interest.

The utility of electrodiagnosis is limited in the acute phase after SCI because it requires Wallerian degeneration to occur before testing will show results; nerve conduction studies require at least 2 weeks, and needle EMG requires at least 4–6 weeks to elapse after injury for changes associated with denervation to reliably present.^20,21^ These tests also require specialized personnel, invasive needle testing, and patient cooperation, which can be especially difficult in those with spasticity.

### Rationale for an magnetic resonance imaging biomarker of lower motor neuron damage

The potential benefits of MRI biomarkers for LMN integrity in SCI are many: LMN viability could be known immediately after injury, allowing for optimal planning and prioritization of treatment efforts; nearly all SCI patients receive an MRI upon first hospital admission, so no additional invasive or costly diagnostic procedures would be required; and acquisition would be independent of the patient's effort, level of consciousness, and cognitive capacity. For our purposes, MR neuroimaging can be split into conventional and quantitative MRI. Conventional MRI encompasses information that can be gained from standard MRI sequences of the injured spinal cord, such as the length and width of the lesion, extent of spinal cord compression, and presence of edema or hemorrhage.

**FIG. 2.**
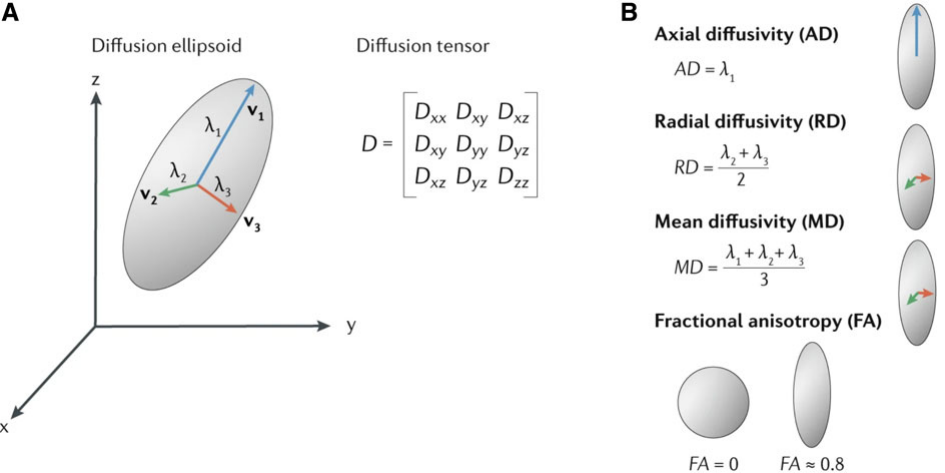
Concepts of diffusion tensor imaging (DTI). (**A**) DTI maps the amount of diffusion in three directions (eigenvalues λ_1_, λ_2_, and λ_3_) to a diffusion tensor. When applied to imaging of neurons, changes in directionality and extent of diffusion can indicate disruptions to the natural highly polarized diffusion parallel to the length of the axon and suggest changes in integrity or functional status. (**B**) From the eigenvalues of DTI, the following parameters are derived: Axial diffusivity is the dominant diffusion direction, typically parallel to the length of the axons; radial diffusivity is diffusion perpendicular to the axial diffusivity. Fractional anisotropy is the degree to which diffusion is restricted to a single direction, as a value between 0 and 1. Mean diffusivity quantifies the overall amount of diffusion occurring between all three planes. Reprinted from *Nature Reviews Neurology*, Vol. 15/12, Gergely David, Siawoosh Mohammadi, Allan R. Martin, Julien Cohen-Adad, Nikolaus Weiskopf, Alan Thompson, and Patrick Freund, Traumatic and nontraumatic spinal cord injury: pathological insights from neuroimaging, pages 718–731, copyright (2019), with permission from Springer Nature.

The most rapidly advancing spinal cord neuroimaging techniques fall under the umbrella of quantitative MRI, which uses special sequences to quantify microstructural features of tissue. The most notable quantitative MRI technique in this context is diffusion tensor imaging (DTI), which assesses changes in the microstructural extent and directionality of water diffusion as indirect measurements of specific pathological processes (Fig. 2). Because diffusion in healthy neurons is naturally polarized (anisotropic), changes in the amount and directionality of diffusion after injury have the potential to identify, localize, and characterize tissue damage earlier and more precisely than currently used diagnostic methods. The metrics of DTI can be analyzed at the voxel level to objectively study specific regions and levels of the spinal cord, for example, the ventral horn and exiting LMNs in the levels within and adjacent to a lesion.^22^

## Methods

To find any published accounts of investigations into either conventional or quantitative MRI utilized to characterize LMN damage in SCI, PubMed, EMBASE, MEDLINE, and the Cochrane Central Register of Controlled Trials were searched on April 27, 2021 for articles published from inception to April 27, 2021 (Supplementary Appendix SA1). Fifty-eight articles were identified after the removal of duplicates. Our inclusion criteria were: 1) The study must use MRI to identify LMN damage, as validated by histological, clinical, or electrodiagnostic exam, and 2) the subject must have a traumatic SCI.

## Results

Two studies were identified. Yang and colleagues^2^ studied 5 patients with SCI and showed that 4 patients with low motor unit count, as estimated by EMG studies, showed MRI evidence of damage to the anterior aspect of the cervical spinal cord, as compared to a control patient with normal motor unit counts, and damage visible on MRI. Frostell and colleagues^23^ found a near-linear (*r* = 0.97) correlation between the length of spinal cord discontinuity, as determined by MRI, and the number of denervated intercostal segments, as determined by EMG, in 5 patients with complete thoracic SCI.

## Discussion

To the extent of our literature search, the application of either conventional or quantitative MRI biomarkers to identify LMN damage remains effectively unexplored. The following discussion therefore attempts to summarize potential MRI biomarkers of spinal cord and neuronal damage that have been examined for other purposes, but may also provide value in future work to characterize a biomarker for LMN damage in SCI.

Conventional MRI features that may be used to predict LMN damage in SCI include longitudinal and axial patterns of hemorrhage, edema, swelling, and compression. Derived from these individual features, the Brain and Spinal Injury Score integrates signals from axial T2-weighted images obtained at the site of the lesion within 48 h of admission to stratify injuries into five patterns based on the extent of hyperintensity and presence of hemorrhage.^24^ This system has shown early validation in predicting American Spinal Injury Association Impairment Scale grade at 1 year, as well as conversion to a lower grade.^25^ Given that these parameters have shown some success in predicting certain neurological outcomes, exploration of potential application to the purpose of identifying LMN damage may be warranted as well.^25–27^

The most useful information from conventional MRI would likely come from a segmentation approach that combines the sagittal length of the lesion with the axial extent into the ventral horn to predict involvement of anterior horn cells at various levels, to better define the injured metamere. Because of the widespread use of conventional MRI in SCI, lack of special image-acquisition techniques required, utility in prognostication of other outcomes, and lack of previous exploration, conventional MRI features could be explored for their utility as potential biomarkers for LMN damage.

Conventional MRI has several potential limitations as a source of biomarker for LMN damage. Interpretation is based solely on identifying areas of hyper- and hypointensity, which are not specific to pathological changes. Further, changes that are below the threshold of visibility are not detected despite potential clinical significance. These limitations can be overcome with new quantitative MRI techniques.

Quantitative MRI techniques such as DTI can detect microstructural tissue integrity to precisely identify damage at the voxel level.^28^ Applying the abundance of data derived from these quantitative MRI techniques shows great promise in the development of early, accurate, and non-invasive biomarkers of LMN integrity. Given that little work on this specific purpose has been published, applications to comparable objectives are examined here through the lens of potential utilization as biomarkers for LMN viability.

DTI uses specialized MRI sequences to quantify the directionality of water diffusion within tissue.^29^ Specific combinations of parameters that indicate the pattern and extent of diffusion within neurons have been recognized to correspond to distinct pathophysiological features of injured spinal cords and peripheral nerves.^30^ DTI has primarily been studied in white matter because it has greater anisotropy; however, gray matter also has an inherent set of natural DTI parameters that change significantly in SCI.^31^ Techniques have been developed to analyze DTI parameters within subdivisions of gray matter using T2*-weighted images to delineate axial regions of interest, overlaid with DTI data.^28,31^ Using this technique, David and colleagues^31^ identified trans-synaptic degeneration specific to the ventral horn of the lumbar enlargement after cervical SCI and correlated DTI parameters of these ventral horns to electrodiagnostic outcomes. This is conceptually promising to the application of defining the injured metamere and specific LMN integrity in the acute phase after SCI, because of the regional specificity and validation to motor function.

The use of DTI in the study of white matter tracts suggests that determining the integrity of LMNs exiting the spinal cord within spinal nerve roots, spinal nerves, plexuses, or peripheral nerves would be feasible in the context of SCI. The idea of applying DTI to the diagnosis of root avulsions is compelling and has been the focus of much recent research, primarily focused on brachial plexus injury.^32^ In the acute phase after traumatic axonal injury, DTI analysis at the site of injury, as well as progressively distal to the lesion over time (because of Wallerian degeneration), shows decreased fractional anisotropy and increased radial diffusivity.^33,34^ These features are less evident proximal to the damaged nerve and more prominent in more-severe injuries, such as axonotmesis or neurotmesis, as compared to neurapraxia.^32,33^ In theory, the high sensitivity for microstructural changes may allow immediate DTI analysis of LMNs to identify peripheral nerve lesions without having to wait for the progression of Wallerian degeneration, as is required in electrodiagnosis.

There are several limitations to DTI that must be overcome before translation into clinical practice for patients with SCI. First, inherent in the technique of DTI is that all measures are indirect and will thus require extensive validation before clinical use. Study of DTI is made even more difficult by the emergent and potentially life-threatening nature of acute SCI given that safety and stabilization take obvious priority over the implementation of advanced imaging techniques. Quantitative MRI spinal cord imaging acquisition is technically challenging because of the small diameter of the spinal cord, physiological motion of the body in the area from the lungs and heart, and presence of metallic stabilization elements.^34^ Overcoming each of these obstacles requires increasing complexity to the hardware, proprietary sequences, and acquisition times.^35^ Although, at present, the challenges described preclude quantitative MRI from meaningful clinical utility, this area is rapidly progressing, and much work is being done to surmount these obstacles and bring these techniques into standard practice.^34^

## Conclusion

As therapies to restore functionality in patients with SCI progress, successful implementation requires awareness of the viability of LMNs. Current methods of LMN diagnosis have limitations that may be overcome by MRI biomarkers. Current literature on the use of conventional or quantitative MRI for LMN diagnosis in SCI is not available. Therefore, we summarized the potential of MRI techniques to this purpose by examining their application to similar scenarios. Our view is that despite limitations preventing immediate translation to clinical practice, investigation into potential utilization of MRI biomarkers to this clinical problem is justified.

## Supplementary Material

Supplemental data

## Supplementary Material

Supplementary Appendix SA1

## Abbreviations Used

DTI: diffusion tensor imaging
EMG: electromyography
LMN: lower motor neuron
MRI: magnetic resonance imaging
SCI: spinal cord injury
UMN: upper motor neurons
