# Supplementary material for: Can Magnetic Resonance Imaging Reveal Lower Motor Neuron Damage after Traumatic Spinal Cord Injury? A Scoping Review
Source: Neurotrauma Rep. 2021 Nov 29;2(1):541–7. doi: 10.1089/neur.2021.0019 (PMC8655802; doi:10.1089/neur.2021.0019)
Supplement: Supplemental data [file Suppl_AppendixSA1.docx]

Supplementary Appendix SA1

**
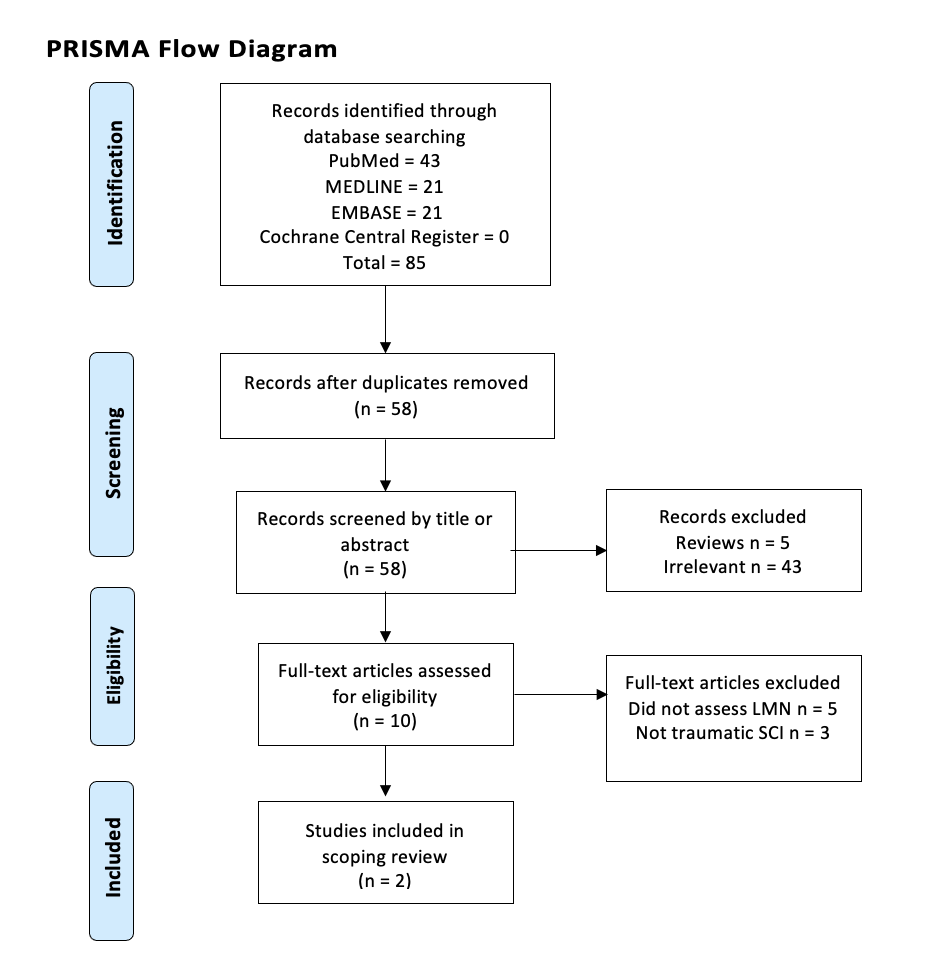
**

**Search Terms:**

**Pubmed**: ("Motor Neurons"[Mesh] OR "Anterior Horn Cells"[Mesh] OR "Peripheral Nerve Injuries"[Mesh] OR “Lower Motor Neuron”) AND ("Spinal cord injuries"[Mesh]) AND ("Magnetic Resonance Imaging"[Mesh] OR "Diffusion Magnetic Resonance Imaging"[Mesh] OR "Multiparametric Magnetic Resonance Imaging"[Mesh] OR "Diffusion Tensor Imaging"[Mesh])

**MEDLINE, EMBASE, Cochrane:** (Spinal Cord Injuries/) AND (magnetic resonance imaging/ or diffusion magnetic resonance imaging/ or multiparametric magnetic resonance imaging/ or diffusion tensor imaging/) AND (motor neurons/ or anterior horn cells/ or peripheral nerve injuries/)
